# Linoleic acid induces human ovarian granulosa cell inflammation and apoptosis through the ER-FOXO1-ROS-NFκB pathway

**DOI:** 10.1038/s41598-024-56970-x

**Published:** 2024-03-16

**Authors:** Wenying Zhang, Fuju Wu

**Affiliations:** Department of Obstetrics and Gynecology, The Second Hospital of Jilin University, Changchun, Jilin China

**Keywords:** Linoleic acid, Granulosa cell, Dietary fatty acids, Polycystic ovary syndrome, Apoptosis, Estrogen receptor, Biochemistry, Cell biology, Molecular biology

## Abstract

Polycystic ovary syndrome (PCOS) is a complex reproductive endocrinological disorder influenced by a combination of genetic and environmental factors. Linoleic acid (LA) is a widely consumed ω-6 polyunsaturated fatty acid, accounting for approximately 80% of daily fatty acid intake. Building upon the prior investigations of our team, which established a connection between LA levels in the follicular fluid and PCOS, this study deeply examined the specific impact of LA using a granulosa cell line. Our findings revealed that LA exerts its influence on granulosa cells (GCs) by binding to the estrogen receptor (ER). Activated ER triggers the transcription of the FOXO1 gene. Reactive oxygen species (ROS)-related oxidative stress (OS) and inflammation occur downstream of LA-induced FOXO1 activation. Increased OS and inflammation ultimately culminate in GC apoptosis. In summary, LA modulates the apoptosis and inflammation phenotypes of GCs through the ER-FOXO1-ROS-NF-κB pathway. Our study provides additional experimental evidence to comprehend the pathophysiology of PCOS and provides novel insights into the dietary management of individuals with PCOS.

## Introduction

Polycystic ovary syndrome (PCOS) affects a significant proportion of women in their childbearing years and manifests as a complex interplay of endocrine and metabolic disorders characterized by hyperandrogenism, anovulation or oligovulation, and polycystic ovary morphology^1–3^. Although a consensus on diagnostic criteria has been globally established in Rotterdam, the Netherlands^4^, the underlying cause of this disease remains elusive. This has resulted in ongoing discussions regarding its pathogenesis. Metabolic aberrations observed in patients with PCOS are extensive, encompassing disruptions in steroid hormone biosynthesis, amino acid and nucleoside metabolism, glutathione metabolism, and lipid and carbohydrates metabolism^5^. Metabolic alterations within the follicular fluid directly affect the surrounding cells, including oocytes and granulosa cells (GCs), consequently influencing ovarian function. Numerous clinical and experimental studies have suggested a critical role of GC apoptosis in the pathogenesis of PCOS, as dysfunctional GCs obstruct folliculogenesis and contribute to follicular atresia at the preantral stage^6–10^. Concurrently, it is widely accepted that patients with PCOS present chronic low-grade inflammation, which is influenced by metabolic status^11,12^. However, whether abnormal metabolites are involved in PCOS pathogenesis by directly influencing GCs remains to be elucidated. A previous analysis of clinical data from our team^13^ and that of Qiao^14^ suggested that there are significant differences in the concentrations of certain fatty acids in the follicular fluid of patients with PCOS compared to infertile women without symptoms of PCOS. Notable variations have been observed in fatty acids such as palmitic acid, linoleic acid (LA), and oleic acid. LA, an essential n-6 polyunsaturated fatty acid (PUFA) in humans, possesses unique characteristics; it is exclusively obtained through diet and serves as a precursor for the synthesis of inflammatory substances, including arachidonic acid and prostaglandin E2. Both studies consistently identified a significant increase in LA levels in patients with PCOS. Therefore, we hypothesized that LA contributes to PCOS progression by influencing ovarian GCs.

## Materials and methods

### Cells treatment

The human ovarian granulosa tumor cell line (KGN) cells were cultivated in DME/F12 (Hyclone) medium. Prior to drug stimulation, 10% DME/F12 medium was replaced with 2% DME/F12 medium. Subsequently, GCs were subjected to LA treatment (75 μM, Sigma-Aldrich) for 24 h, with or without a 1 h pre-treatment of the estrogen receptor (ER) antagonist ICI 182780 (0.05 μM, MCE), the forkhead box O 1 (FOXO1) inhibitor AS1842856 (1 μM, MCE), N‐Acetyl‐cysteine (NAC) (2 mM, Beyotime Biotechnology, China), and the NF-κB inhibitor JSH-23 (4 μM, MCE). LA was dissolved in PBS and NAC was dissolved in sterile water, whereas ICI 182780, AS 1842856, and JSH-23 were dissolved in DMSO. The controls received only their respective vehicle.

### Cell counting kit-8 (CCK-8) test

GCs were seeded (100 μL/well) in a 96-well plate, followed by a 24 h pre-cultivation period (at 37 °C, 5% CO_2_). Subsequently, different concentrations of LA (50, 75, and 100 μM) were added to the culture medium, stimulating the GCs for an additional 24 h. Next, 10 μL of CCK solution was added to each well, and the culture plate was further incubated in the incubator for 4 h. The absorbance at 450 nm was measured using a microplate reader.

### Enzyme-linked immunosorbent assay (ELISA)

Culture supernatants from each group were collected to assess the concentrations of the inflammatory cytokines interleukin-6 (IL-6), interleukin-1β (IL-1β), and tumor necrosis factor α (TNF-α) using ELISA kits from Proteintech. All procedures were performed in strict accordance with manufacturer’s instructions. Absorbance was measured using a microplate reader at 450 nm, and the results were analyzed using ELISACalc software.

### Real-time quantitative reverse transcription-polymerase chain reaction (qRT-PCR)

Initially, total RNA was extracted according to the procedural guidelines available on the official website of Takara Biomed. Subsequently, RNA was reverse-transcribed to cDNA using the Takara cDNA Synthesis Kit. Finally, a qRT-PCR system was employed, consisting of 10 μL SYBR, 0.5 μL forward primer, and 0.5 μL reverse primer for the target gene, along with 4.6 μL ddH_2_O. The qRT-PCR was carried out at 95 °C for 3 min, followed by 40 cycles of 60 °C for 60 s and 95 °C for 15 s, to determine the relative mRNA expression levels of IL-6, IL-1β, TNF-α, CASPASE3, estrogen receptor 1(ESR1), estrogen receptor 2 (ESR2), FOXO1, and nuclear factor kappa-B 1 (NFκB1). Data analysis was performed using the 2^−△△Ct^ method. Primer sequences for the aforementioned genes are listed in Table 1.

**Table 1:** Sequence list of primers used for qRT-PCR.

Gene	Sequence of forward primer	Sequence of reverse primer
GAPDH	5’-ATTTGGCTACAGCAACAGG-3’	5’-TTGAGCACAGGGTACTTTATT-3’
CASPASE3	5’-CTGGACTGTGGCATTGAGAC-3’	5’-GCAAAGGGACTGGATGAACC-3’
IL-6	5’-ACTCACCTCTTCAGAACGAATTG-3’	5’-CCATCTTTGGAAGGTTCAGGTTG-3’
IL-1β	5’-CAGAAGTACCTGAGCTCGCC-3’	5’-AGATTCGTAGCTGGATGCCG-3'
TNF-α	5’-GCAACTGCTGCACGAAATC-3’	5’-CTGCTTGTCCTCTGCCCAC-3'
ESR1	5’-GAAAGGTGGGATACGAAAAGACC-3’	5’-GCTGTTCTTCTTAGAGCGTTTGA-3’
ESR2	5’-TCCATCGCCAGTTATCACATCT-3’	5’-CTGGACCAGTAACAGGGCTG-3’
FOXO1	5’-TCGTCATAATCTGTCCCTACACA-3’	5’-CGGCTTCGGCTCTTAGCAAA-3’
NFκB1	5’-AACAGAGAGGATTTCGTTTCCG-3’	5’-TTTGACCTGAGGGTAAGACTTCT-3’

*GAPDH* Glyceraldehyde-3-phosphate dehydrogenase, *CASPASE3* Cysteine-aspartic acid protease 3, *IL-6* Interleukin-6, *IL-1β* Interleukin-1β, *TNF-α* Tumor necrosis factor α, *ESR1* Estrogen receptor 1, *ESR2* Estrogen receptor 2, *FOXO1* Forkhead box O 1, *NFκB1* Nuclear factor kappa-B 1.

### Caspase 3 activity assay

After being treated as described above, GCs were collected by trypsin. Subsequent procedures were strictly following manufacturer’s instructions by using Caspase 3 Activity Assay Kit (Beyotime Biotechnology, China). The absorbance of end-products was measured using a microplate reader at 405 nm.

### Western blotting (WB)

Following protein extraction from the cellular lysate and quantification using a BCA protein assay kit (Beyotime Biotechnology, China), the samples were subjected to sodium dodecyl sulfate–polyacrylamide gel electrophoresis (SDS-PAGE). The separated protein bands were transferred to PVDF membranes. Primary antibodies against FOXO1 (1:1,000, Proteintech), NF-κB subunit p65 (1:1,000, Proteintech), phospho-NF-κB p65 ((1:1,000, Abmart), IκBα (1:1,000, Cell Signaling Technology), phospho-IκBα (1:1,000, Cell Signaling Technology), and glyceraldehyde-3-phosphate dehydrogenase (GAPDH, 1:5,000, Proteintech) were incubated with the PVDF membranes at 4 °C overnight, followed by binding with an HRP-conjugated secondary antibody for 2 h at room temperature. The final signals were visualized using an enhanced chemiluminescence substrate kit. The original WB images are displayed in the Supplementry material.

### Flow cytometry

Apoptosis and cellular levels of reactive oxygen species (ROS) were measured by flow cytometry. Various pretreatments were administered to the stimulated cells using the Annexin V-FITC apoptosis detection kit (Beyotime Biotechnology, China) and the ROS assay kit (Beyotime Biotechnology, China). Live cells loaded with appropriate probes were analyzed using a BD FACSCalibur flow cytometer.

### Molecular docking

First, the structure-data file (SDF) of the ligand was retrieved from the PubChem database. Subsequently, the obtained SDF file was converted into a Protein Data Bank (PDB) file format using OpenBabel. In the second step, the AutoDock Tools 1.5.6 software was utilized for preprocessing tasks, including dehydration and hydrogenation, on the protein targets. Furthermore, both the active ingredients and target protein structures were transformed into pdbqt format to facilitate subsequent molecular docking studies. Finally, molecular docking analyses were conducted using AutoDock Vina, allowing for a comprehensive exploration of the binding interactions between the ligands and protein targets.

### Molecular dynamics simulation

First, a molecular system was defined by specifying the molecules and their initial configurations. Next, an appropriate force field was selected to describe the intermolecular interactions. Energy minimization was performed to relax the system, followed by equilibration to adjust the temperature and pressure. The main production run was initiated, allowing the system to evolve over time, and trajectory data were recorded. The root mean square deviation (RMSD) was used to characterize the behavior of the system.

### Statistical analysis

All experiments were independently repeated at least 3 times. Comparisons between two groups were assessed using the Independent-Samples T Test, whereas multiple comparisons were analyzed using one-way ANOVA with Tukey’s post-hoc test. Data were presented as mean ± SEM. GraphPad Prism (version 8.0) was used to create bar and line charts. Statistical significance was set at *P* < 0.05.

## Results

### LA causes GC apoptosis and inflammation

**Figure 1:**
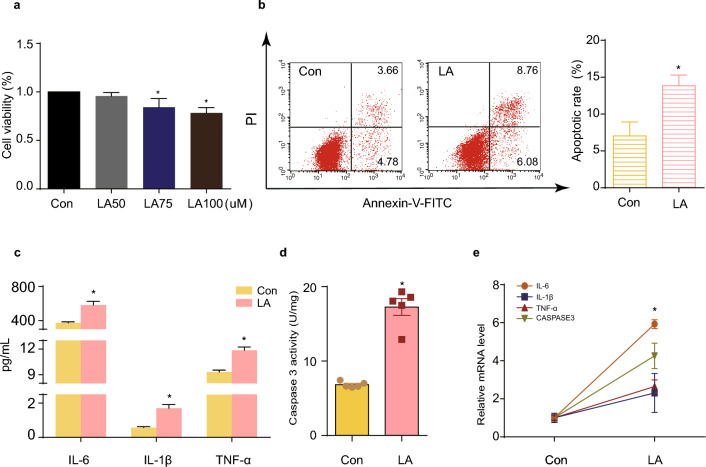
LA causes GC apoptosis and inflammation. (**a**) Dose-dependent effect of LA on GC viability. N = 3. (**b**) Apoptotic effect of LA on KGN. N = 3. (**c**) LA stimulated the secretion of inflammatory cytokines IL-6, IL-1β, and TNF-α in GCs. N = 3. (**d**) LA increased intracellular CASPASE3 protein activity. N = 5. (**e**) Real-time PCR results showing IL-6, IL-1β, TNF-α, and CASPASE3 mRNA levels after LA addition. N = 3. *P < 0.05 versus control group, ^#^*P* < 0.05 versus LA group. CON: control group; LA: LA group; IL-6: interleukin-6; IL-1β: interleukin-1β; TNF-α: tumor necrosis factor α; CASPASE3: cysteine-aspartic acid protease 3.

Prior to the phenotypic observations, we determined the optimal concentration of LA using the CCK-8 assay (Fig. 1a). Various concentrations of LA (50, 75, and 100 μM) were added to the cell culture medium for 24 h. Notably, 50 μM LA exhibited no apparent impact on cell viability, whereas 75 μM LA inhibited the activity of GCs, and 100 μM significantly decreased cell viability. To minimize drug toxicity, we selected the 75 μM concentration for subsequent experiments.

Following the addition of LA, the rate of apoptosis in KGN cells increased significantly (Fig. 1b), and both the mRNA levels (Fig. 1e) and activity (Fig. 1d) of CASPASE 3 were notably elevated. In terms of inflammation, LA significantly stimulated the secretion of IL-6, IL-1β, and TNF-α by KGN (Fig. 1c), evidenced by increased corresponding mRNA levels (Fig. 1e).

### LA affects GC apoptosis and inflammation through ER

**Figure 2:**
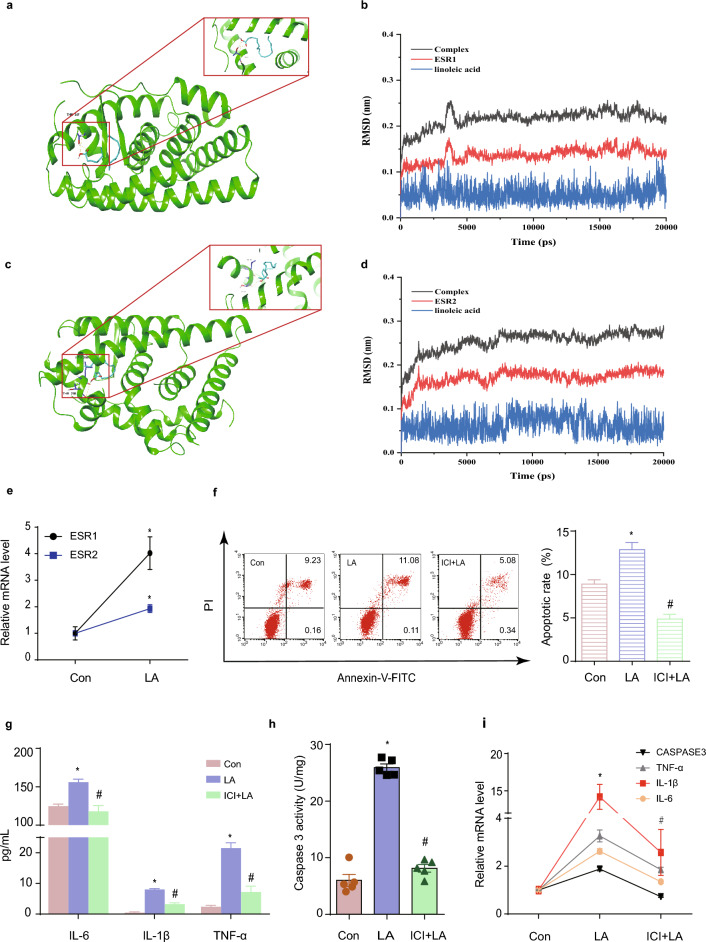
LA affects GC apoptosis and inflammation through the ER. (**a**) The molecular docking results of LA and ESR1 with a binding energy of − 6.9 kcal/mol. (**b**) The molecular dynamics simulation results for the interaction between LA and ESR1 revealed a RMSD fluctuation range of their complex within 0.02. (**c**) The molecular docking result of LA and ESR2 with a binding energy of -6.1 kcal/mol. (**d**) The molecular dynamics simulation results for the interaction between LA and ESR2 revealed that the RMSD fluctuation range of their complex is within 0.03. (**e**) Real-time PCR confirmed the increase of ESR1 and ESR2 mRNA levels after LA treatment. N = 3. (**f**) ER antagonist ICI 182780 decreased the apoptosis rate of GCs induced by LA. N = 3. (**g**) The hypersecretion of IL-6, IL-1β, and TNF-α was inhibited by ICI 182780. N = 3. (**h**) CASPASE3 activity was suppressed in the LA + ICI 182780 group compared with that in the LA group. N = 5. (**i**) mRNA levels of IL-6, IL-1β, TNF-α, and CASPASE3 after treatment of LA in the absence or presence of ICI 182780. N = 3. *P < 0.05 versus control group, ^#^*P* < 0.05 versus LA group. CON: control group; LA: LA group; ICI + LA: ICI 182780 + LA group; RMSD: root mean square deviation; IL-6: interleukin-6; IL-1β: interleukin-1β; TNF-α: tumor necrosis factor α; CASPASE3: cysteine-aspartic acid protease 3.

From our prophase RNA-seq results, ER-related genes differentially expressed in LA-treated GCs. In this study, we confirmed that treatment of KGN with LA resulted in ESR1 and ESR2 upregulation (Fig. 2e). After 50 rounds of molecular docking, it was observed that the binding energy between LA and ESR1 reached a minimum value of − 6.9 kcal/mol (Fig. 2a). Similarly, the binding energy between LA and ESR2 was determined to be as low as − 6.1 kcal/mol (Fig. 2c). The results of the molecular dynamics simulations suggested that LA is capable of establishing stable complexes with ESR1 and ESR2. Specifically, the RMSD fluctuation range for the complex formed with ESR1 was within 0.02, whereas the RMSD fluctuation range for the complex formed with ESR2 was within 0.03. (Fig. 2b, d). Next, we pretreated the cells with the ER antagonist ICI 182,780. This led to a notable reduction of apoptosis in KGN cells (Fig. 2f), accompanied by a significant decrease in both mRNA level and activity of CASPASE 3 (Fig. 2h, i). Additionally, the inflammation of KGN markedly improved, as evidenced by the reduced secretion of inflammatory factors IL-6, IL-1β, and TNF-α (Fig. 2g) and the decrease in their respective mRNA levels (Fig. 2i).

### LA activates FOXO1 expression through the ER

**Figure 3:**
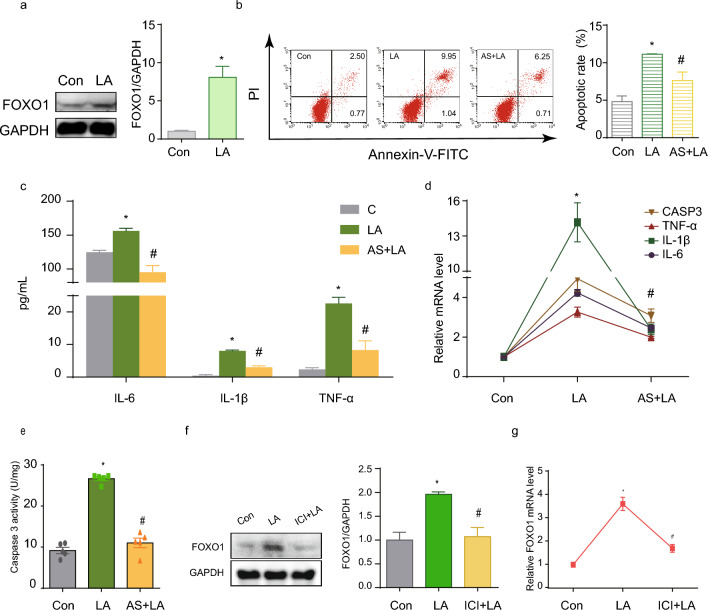
LA activates FOXO1 expression through the ER. (**a**) The WB results suggested FOXO1 activation by LA. (**b**) The FOXO1 inhibitor AS1842856 reduced the high apoptosis rate of GCs caused by LA. N = 3. (**c**) ELISA revealed that the level of inflammatory cytokines returned to values comparable to baseline after adding FOXO1 inhibitor AS1842856. N = 3. (**d**) Corresponding mRNA levels of IL-6, IL-1β, TNF-α, and CASPASE3 in the absence or presence of AS1842856. N = 3. (**e**) Effect of LA on CASPASE3 activity with or without AS1842856. N = 5. (**f**, **g**), WB and real-time PCR results suggested that the increased FOXO1 level returned to values comparable to baseline when the effect of LA was antagonized by ICI 182,780. N = 3. *P < 0.05 versus control group, ^#^*P* < 0.05 versus LA group. CON: control group; LA: LA group; AS + LA: AS1842856 + LA group; GAPDH: glyceraldehyde-3-phosphate dehydrogenase; IL-6: interleukin-6; IL-1β: interleukin-1β; TNF-α: tumor necrosis factor α; CASPASE3: cysteine-aspartic acid protease 3; FOXO1: forkhead box O 1.

To identify the downstream molecular mechanism behind LA triggered ER activation on GCs, we focused on FOXO1, which has been reported as a down-stream gene involved in ER activation and it plays a critical role in many cell signaling pathways, such as oxidative stress (OS), inflammation, apoptosis. WB showed an increase in FOXO1 protein expression after LA treatment (Fig. 3a). Consequently, we used the FOXO1 inhibitor AS1842856 to investigate whether FOXO1 is involved in LA effect on GCs. Upon inhibition of FOXO1, the previously elevated apoptosis rate was markedly decreased (Fig. 3b). This reversal was further evidenced by a decrease in mRNA expression (Fig. 3d) and the protein activity of the apoptosis-related gene CASPASE3 (Fig. 3e). Additionally, the heightened secretion of inflammatory factors IL-6, IL-1β, and TNF-α demonstrated a decrease, as confirmed by both protein (Fig. 3c) and mRNA levels (Fig. 3d). To clarify whether the ER is involved in the regulation of FOXO1, we examined FOXO1-related indicators. Blocking ERs resulted in the normalization of the FOXO1 protein expression (Fig. 3f), accompanied by a noticeable decrease in its elevated mRNA levels (Fig. 3g).

### LA leads to intracellular ROS rise by ER-FOXO1 pathway

**Figure 4:**
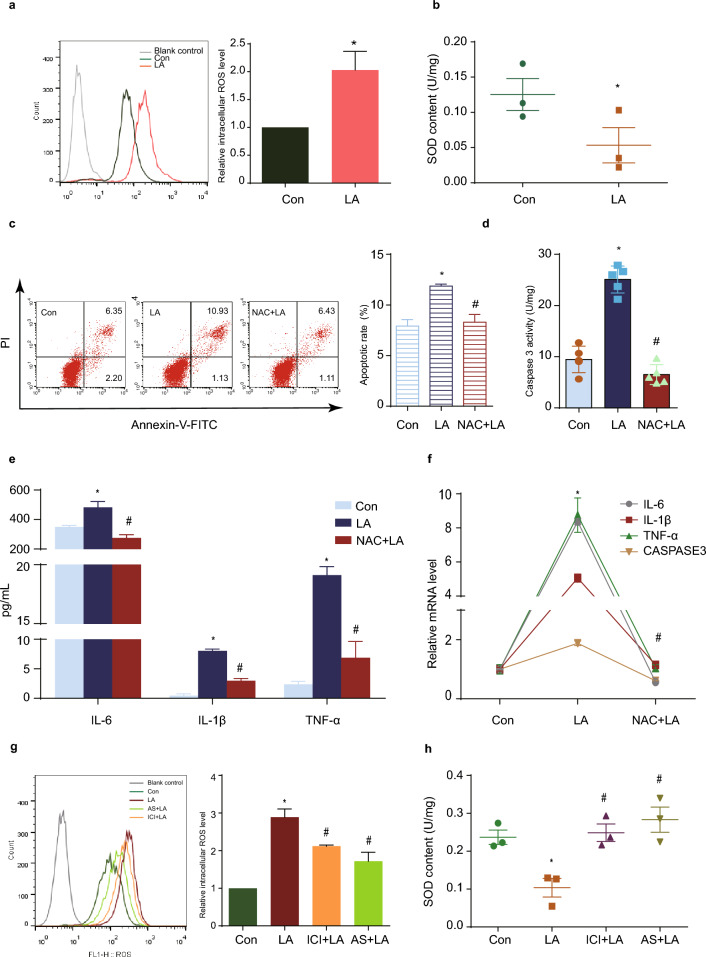
LA leads to increased intracellular ROS levels through the ER-FOXO1 pathway. (**a**) Flow cytometry analyses showed that intracellular ROS was significantly increased. N = 3. (**b**) SOD content was reduced by LA. N = 3. (**c**) The apoptosis rate was reduced after administration of NAC. N = 3. (**d**) NAC enabled CASPASE3 activity level to normal. N = 5. (**e**) The excessive secretion of inflammatory cytokines IL-6, IL-1β, and TNF-α were hindered by NAC. N = 3. (**f**) The RNA expression of IL-6, IL-1β, TNF-α, and CASPASE3 decreased with NAC addition. N = 3. (**g** and **h**) Pre-treating KGN with ICI 182,780 and AS1842856 abolished the LA-induced intracellular changes in both ROS and SOD. N = 3. *P < 0.05 versus control group, ^#^*P* < 0.05 versus LA group. CON: control group; LA: LA group; ICI + LA: ICI 182,780 + LA group; AS + LA: AS1842856 + LA group; NAC: N‐Acetyl‐cysteine; GAPDH: glyceraldehyde-3-phosphate dehydrogenase; IL-6: interleukin-6; IL-1β: interleukin-1β; TNF-α: tumor necrosis factor α; CASPASE3: cysteine-aspartic acid protease 3; SOD: superoxide dismutase.

It is well known that OS is an important manifestation in cellular and organ damages^15^. To investigate the role of LA regulating OS, we measured the intracellular ROS generation and cellular antioxidant capacity. We noticed a marked increase in ROS intracellular levels in LA-treated KGN (Fig. 4a), accompanied by a decrease in the levels of the antioxidant enzyme superoxide dismutase (SOD) (Fig. 4b). When KGN cells received NAC before LA treatment, apoptosis- and inflammation-related indicators showed appreciable recovery. NAC alleviated GCs apoptosis (Fig. 4c), resulting in decreased CASPASE3 activity (Fig. 4d) and mRNA expression (Fig. 4f). Furthermore, NAC hampered the excessive secretion of IL-6, IL-1β, and TNF-α (Fig. 4e, f). Subsequently, we investigated whether the ER-FOXO1 pathway mediates the regulation of ROS changes. ICI 182780 and AS1842856 suppressed the increase in ROS intracellular levels (Fig. 4g) and concomitantly restored SOD ones (Fig. 4h).

### LA induces NF-κB expression through the ER-FOXO1-ROS pathway

**Figure 5:**
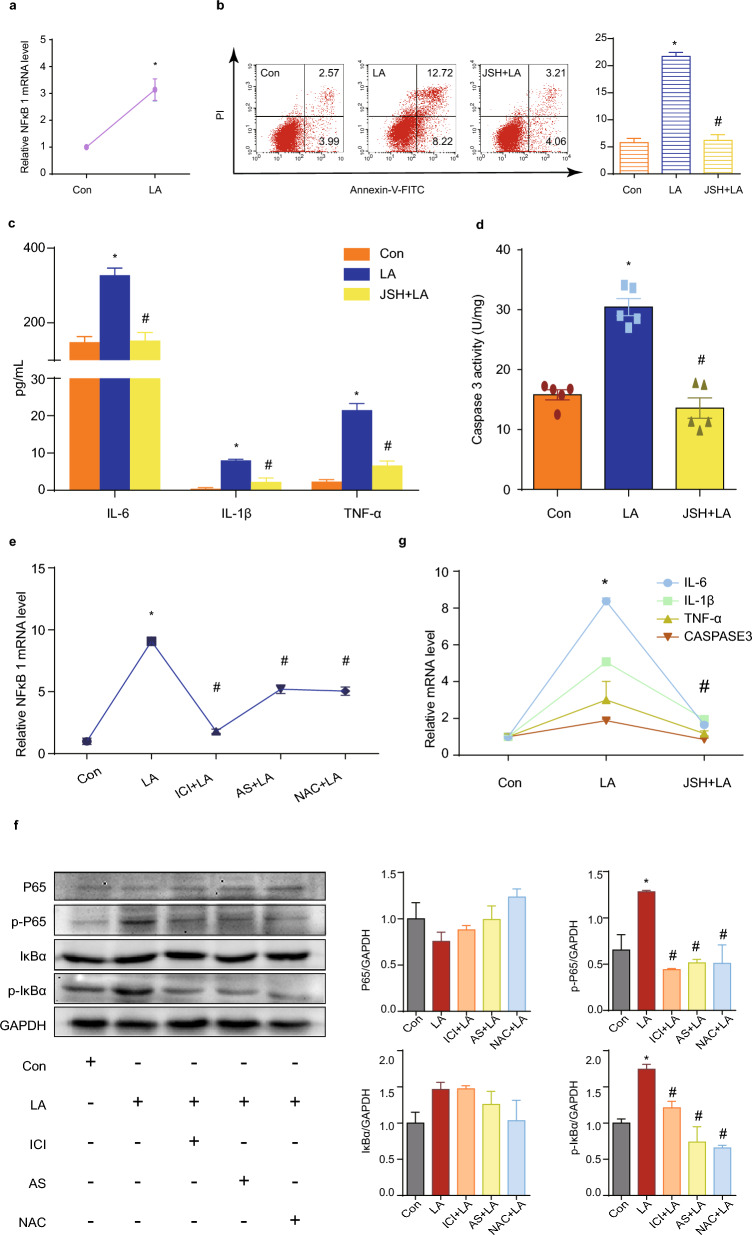
LA induces NF-κB expression through the ER-FOXO1-ROS pathway. (**a**) LA induced the expression of NFκB mRNA 1. N = 3. (**b**) JSH-23 reversed the apoptotic effect induced by LA. N = 3. (**c**) Concentrations of inflammatory cytokines were restored by JSH-23. N = 3. (**d**) CASPASE3 activity measurement in the absence and presence of JSH-23. N = 5. (**e** and **f**) Due to LA increased the protein level of p-P65 and p-IκBα and the mRNA level of NFκB 1, it enhanced GCs inflammation through the ER-FOXO1-ROS pathway. N = 3. (**g**) mRNA expressions of IL-6, IL-1β, TNF-α, and CASPASE3 in the presence or absence of JSH-23. N = 3. *P < 0.05 versus control group, ^#^*P* < 0.05 versus LA group. CON: control group; LA: LA group; JSH + LA: JSH-23 + LA group; ICI: ICI 182,780; AS: AS1842856; NAC: N‐Acetyl‐cysteine; GAPDH: glyceraldehyde-3-phosphate dehydrogenase; IL-6: interleukin-6; IL-1β: interleukin-1β; TNF-α: tumor necrosis factor α; CASPASE3: cysteine-aspartic acid protease 3; FOXO1: forkhead box O 1; P65: NF-κB subunit p65;p-P65: phosphorylated NF-κB subunit p65; IκBα: NF-κB inhibitor alpha ; p-IκBα: phosphorylated NF-κB inhibitor alpha.

OS is regarded as one of triggers of the inflammation response, in which NF-κB serves as a pivotal mediator^16^. We found that in GCs, NF-κB 1 (p50) was up-regulated after LA stimulation at the mRNA level (Fig. 5a). The use of the NF-κB inhibitor JSH-23 significantly repressed the observed increase in apoptosis, as indicated by the apoptotic rate (Fig. 5b) and CASPASE3 levels (Fig. 5d, g). The secretion of inflammation factors IL-6, IL-1β and TNF-α was prevented by JSH-23 (Fig. 5c, g). The relationship of ROS and NF-κB depends on different upstream pathways and specific cell^17^. Our further research aimed to dissect the upstream regulation of NF-κB. By blocking ER, repressing FOXO1, and clearing excess intracellular ROS, LA was unable to elevate mRNA expression of NF-κB 1 (Fig. 5e). More intuitively, LA enhanced the protein expressions of phosphorylated IκBα and phosphorylated NF-κB subunit p65, whereas corresponding inhibitors eliminated the enhancement in these protein expressions (Fig. 5f).

## Discussion

LA is an important PUFA and its deficiency has been linked to sterility^18^. However, LA derivatives, such as hydroxyoctadecadiene acids (HODE), play a role in pathological conditions related to reproduction, especially PCOS^19–21^. No research has been conducted on the correlation between reproductive diseases and LA itself. Based on our discovery of a significant difference in the concentration of LA in the follicular fluid of patients with PCOS^13^, this study further demonstrated that LA induces ovarian GCs apoptosis and inflammation, potentially contributing to the etiology of PCOS. RNA-seq analysis of PCOS GCs has revealed that differentially expressed genes (DEGs) in lipid metabolism pathway and fatty acid biosynthetic processes are enriched (DEGs numbers of the two pathways are more than that of other GO or KEGG pathways) and abnormally expressed^22^. During follicular development, the intrafollicular cells of polycystic ovaries exhibit abnormal maturation, leading to the absence of dominant follicles, which is a principal reason for anovulation and oligovulation. As the largest number of supporting cells in follicles, ovarian GCs not only provide nutrients and growth regulators for oocyte development but also determine the fate of follicles^23^. A popular viewpoint in the academic community is that apoptosis of GCs may be involved in ovulatory dysfunction in PCOS^6–8,24^. Although apoptosis of ovarian GCs tends to increase according to the stage of follicular development^25^, patients with PCOS exhibit high percentages of both early and late apoptosis^6^, along with proliferation suppression^26^. The GCs of androstenedione-induced PCOS mice not only showed increased apoptosis but also exhibited abundant lipid droplets^27^, which is consistent with our results. The apoptosis-inducing effect of LA has also been demonstrated in colorectal cancer^28^ and human aortic endothelial cells^29^. Additionally, high-dose linoleic was shown to inhibit the viability of rat pancreatic exocrine cells^30^.

ER is a ligand-activated protein belonging to the steroid and nuclear receptor superfamily^31^. The ER regulates the transcription of genes involved in growth, metabolism, sexual development, gestation, and other reproductive functions^32^. Mammalian ER is encoded by two genes, ESR1 and ESR2, which function as signal transducers and transcription factors to modulate the expression of target genes^31^. The molecular docking and molecular dynamics simulations conducted in our study collectively suggested that LA exhibits a consistent and stable binding affinity for ERs. Thus, it is a potential ligand that binds to both ER subtypes, thereby regulating the expression of downstream genes. Following LA treatment, the mRNA expression of these two genes in GCs significantly increased. Blockade of ER by ICI 182780 resulted in the abrogation of apoptosis induced by LA, confirming that binding to ER is the initial step in LA effect on GCs. In 1998, Hilakivi-Clarke et al. confirmed that a high-fat diet (HFD) may be an important factor in increasing estrogenic activity during pregnancy^33^. Different dietary PUFAs may affect lipid metabolism by altering ER expression^34^. In a case-case study, investigators discovered that dietary consumption of LA influenced the ER status in patients with premenopausal breast cancer^35^. The alterations in the expression of ERα and ERβ in PCOS may be related to abnormal follicular development^36^. A mouse model showed that excessive ER activation by injecting estrogen into female mice can cause anovulation and follicular cysts^37^. More importantly, ERβ may play a role in the pathogenesis of GC tumor, acting as a binding partner of proteins involved in the apoptotic cascade in GCs^38^.

FOXO1 belongs to the forkhead transcription factor (FOX) family, which modulates various downstream genes involved in apoptosis, autophagy, OS, cell cycle, and metabolic and immune regulation^39^. In our study, the activation of FOXO1 by LA increased intracellular ROS levels, indicating a crucial role for FOXO1 in mediating the oxidative and anti-oxidative systems in GCs. When AS1842856 was used to repress FOXO1, the LA-induced changes in GCs were fully restored. LA binds to the ER to activate FOXO1, inducing an increase in the transcription and expression of FOXO1. This is the first study to report the relationship between LA and FOXO1 expression. Consistent with our findings, non-esterified fatty acids induce OS, apoptosis, and steroid hormone synthesis disorders in bovine GCs by regulating FOXO1 phosphorylation and nuclear translocation^40^. Additionally, FOXO1 has been implicated in mediating heparin-binding epidermal growth factor-like growth factor (HB-EGF) -induced apoptosis of GCs^41^.

OS-induced injury in GCs is considered a common trigger for follicular atresia^42^. Women with PCOS have increased OS and decreased anti-oxidant capacity^43^. OS in cells manifests as an over-physiological level of ROS. Elevated ROS expression levels in PCOS GCs significantly induce cell apoptosis, thereby affecting oocyte quality and reducing the positive outcomes of in vitro fertilization-embryo transfer (IVF-ET) in women with PCOS^44^. We confirmed that LA induces ROS production in GCs, accompanied by a decrease in the antioxidative enzyme SOD. This phenomenon was attributed to increased expression of FOXO1. SOD catalyzes the dismutation of O_2_¯ to H_2_O_2_ and O_2_. The anti-oxidant capacity eliminated by LA was restored by the ER antagonist ICI 182780 and FOXO1 inhibitor AS1842856, further the apoptotic and inflammatory phenotypes were also rescued by the inhibitors. The protective effects of ameliorating OS and ROS by inhibiting FOXO1 were observed in primary rat alveolar epithelial cells subjected to smoke inhalation-induced lung injury^45^. LA-induced OS has also been identified in tissues such as the liver of cows^46^.

Numerous clinical data have explored the associations between LA and inflammation, but conflicting opinions persist regarding whether it increases or decreases inflammation^47,48^. Our results unequivocally confirm that LA exposure induces inflammation in ovarian GCs. Consistent with our findings, a HFD rich in LA was linked to an increased risk of colon inflammation^49^. PCOS is a chronic, low-grade inflammatory disease^50^. Infertile women with PCOS have higher TNF-α and IL-6 levels in follicular fluid^51,52^, and elevated IL-1β level in serum^53^. We determined that LA-induced phosphorylation of the NF-κB subunit p65 and IκBα, activating the canonical NF-κB pathway, stimulated the secretion of pro-inflammatory cytokines IL-6, IL-1β, and TNF-α by GCs, providing a possible explanation for the surge in their levels. Simultaneously, the expression of another NF-κB subunit, NF-κB 1 (p105/p50), was enhanced. The p50-p65 heterodimer plays a pivotal role in the canonical NF-κB pathway^54^. Under normal conditions, these dimers are retained in the cytoplasm, bound by IκB^55^. Activation of the canonical NF-κB pathway involves phosphorylation of IκBα, leading to the release of the p50-p65 dimer and phosphorylation of the p65 subunit. This process facilitates translocation of the heterodimer to the nucleus and activates target gene transcription. Phosphorylated p65 is crucial for the interaction of RelA with CBP/300 coactivator complexes, enabling its translocation from the cytoplasm to the nucleus, activating the NF-κB pathway^56^, resulting in histone and p65 acetylation, and promoting target gene transcription^57^. Our findings demonstrate that LA activates the canonical NF-κB pathway in GCs. Adding NAC to scavenge ROS hinders the phosphorylation of p65 and IκBα, as well as the secretion of pro-inflammatory cytokines, implying that ROS is upstream of NF-κB^58^. ROS has been shown to activate NF-κB through alternative IκBα phosphorylation^17^. Respectively inhibiting ER and FOXO1, and clearing intracellular ROS obstructed the activation of NF-κB by LA, and subsequent apoptosis. Recently, LA has been reported to affect the apoptosis pathway through NF-κB in in vitro development of parthenogenic porcine embryos^59^. As found in our research, the NF-κB inhibitor JSH-23 can block LA-induced GCs apoptosis, indicating that NF-κB mediate LA-induced apoptosis.

A previous study indicated that patients with PCOS have a higher percentage of apoptotic GCs than the control group^6^. Increased apoptosis in GCs has been identified as a major factor contributing to aberrant follicle maturation^7^, underlying the abnormal ovarian function observed in PCOS. Regardless of whether apoptosis is initiated extracellularly or intracellularly, the caspase cascade serves as the terminal phase of apoptosis^60^. Caspase-3, the ultimate effector, contributes to apoptosis by directly disassembling cell structures^61^. Boone et al. reported that Caspase-3 is localized in the GCs of atretic follicles rather than in healthy follicles^62^. Our work demonstrated that the level of CASPASES3 was increased and its activity was enhanced by LA, in accordance with the observed apoptotic effect. NF-κB inhibitor JSH-23 impeded the activation of CASPASE3, further confirming that the activation of NF-κB is a pre-requisite for LA-induced GCs apoptosis. Prior to incubation with LA, pre-treatment respectively with ICI 182780, AS 1842856, and NAC reduced the apoptotic rate of GCs, revealing that LA-induced GCs apoptosis is mediated by ER-FOXO1-ROS- NF-κB pathway.

## Conclusion

**Figure 6:**
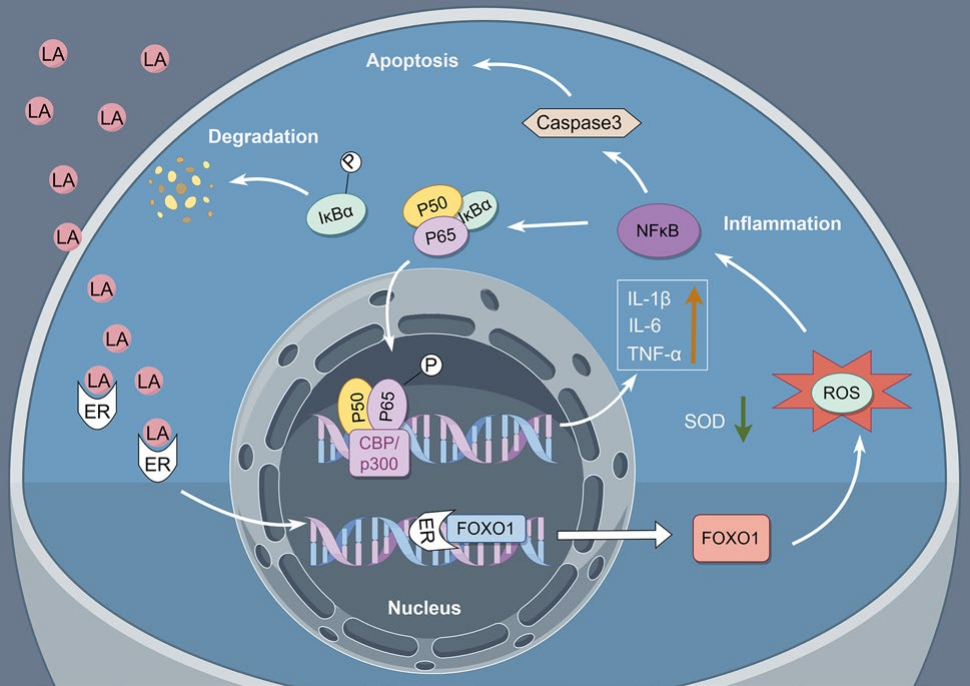
Schematic diagram illustrating the regulatory mechanism of LA on GC apoptosis and inflammation. LA exerts its effect by binding to the ER and subsequently initiating the activation of FOXO1. Elevated FOXO1 expression results in a redox system imbalance, manifested as increased ROS and decreased SOD levels. This imbalance further activates inflammation-related indicators. The p50/p65 complex uncouples with IκBα, then p65 and IκBα are phosphorylated. p-IκBα undergoes degradation, whereas p-P65 translocates to the nucleus to stimulate downstream NFκB-related gene expression such as IL-6, IL-1β, and TNF-α. Simultaneously, the activation of NFκB leads to CASPASE3 overexpression and enhances its activity, ultimately resulting in GC apoptosis. (Constructed using Figdraw) LA: linoleic acid; ER: estrogen receptor; FOXO1: forkhead box O 1; ROS: reactive oxygen species; NFκB: nuclear factor kappa-B; SOD: superoxide dismutase; IL-6: interleukin-6; IL-1β: interleukin-1β; TNF-α: tumor necrosis factor α; CASPASE3: cysteine-aspartic acid protease 3; P65: NF-κB subunit p65; p-P65: phosphorylated NF-κB subunit p65; IκBα: NF-κB inhibitor alpha ; p-IκBα: phosphorylated NF-κB inhibitor alpha; CBP/p300: histone acetyltransferases CBP and p300.

Our study showed that LA can induce apoptosis and inflammation in GCs through the ER-FOXO1-ROS-NF-κB pathway (Fig. 6). To the best of our knowledge, this is the first study to identify the precise pathway through which LA affects GCs, with experimental data confirming that ER is the target receptor of LA. As the detrimental effects of LA on GCs, this type of ω-6 PUFAs may participant in the pathophysiology of PCOS, which should draw more attention. Given that the present dietary recommendation is to consume higher ratio of ω-6 PUFAs to lower the incidence of cardiovascular diseases, it is imperative to be more vigilant about the reproductive risks associated with this type of dietary choices. Appropriate management of inflammation and apoptosis in GCs is critical for regaining ovarian function in patients with PCOS. Our research has enabled a deeper understanding of the pathophysiological mechanisms underlying PCOS. In order to collect more data confirming the clinical phenotypes of PCOS patients, our team is currently conducting animal experiments on LA administration.

### Supplementary Information

Supplementary Information.

## Data Availability

All data generated or analyzed during this study are included in this published article.

## Abbreviations

PCOS: Polycystic ovary syndrome
GCs: Granulosa cells
LA: Linoleic acid
PUFA: N-6 polyunsaturated fatty acid
ER: Estrogen receptor
FOXO1: Forkhead box O 1
NAC: N‐acetyl‐cysteine
IL-6: Interleukin-6
IL-1β: Interleukin-1β
TNF-α: Tumor necrosis factor α
qRT-PCR: Real-time quantitative reverse transcription-polymerase chain reaction
ESR1: Estrogen receptor 1
ESR2: Estrogen receptor 2
NF-κB: Nuclear factor kappa-B
SDS-PAGE: Sodium dodecyl sulfate-polyacrylamide gel electrophoresis
ROS: Reactive oxygen species
SDF: Structure-data file
PDB: Protein data bank
RMSD: Root mean square deviation
SOD: Superoxide dismutase
HODE: Hydroxyoctadecadiene acids
HFD: High-fat diet
OS: Oxidative stress
IVF-ET: In vitro fertilization-embryo transfer
DEGs: Differentially expressed genes
GO: Gene ontology
KEGG: Kyoto Encyclopedia of Genes and Genomes
HB-EGF: Heparin-binding epidermal growth factor-like growth factor
