# Supplementary material for: Linoleic acid induces human ovarian granulosa cell inflammation and apoptosis through the ER-FOXO1-ROS-NFκB pathway
Source: Sci Rep. 2024 Mar 16;14:6392. doi: 10.1038/s41598-024-56970-x (PMC10944505; doi:10.1038/s41598-024-56970-x)
Supplement: Supplementary file 1 — Supplementary Information. [file 41598_2024_56970_MOESM1_ESM.pdf]

Linoleic acid induces human ovarian granulosa cell inflammation and apoptosis through the ER-FOXO1-ROS-NFκB pathway

Wenying Zhang[1], Fuju Wu[1]*

Raw data of WB

We declare and ensure that our WB data comply with the digital image and integrity policies. All images were unchanged (Only cropped). The PVDF membranes were cut based on molecular weight of targeted protein before hybridization with a single specific target antibody during blotting for better imaging. For some antibodies with poor specificity and more miscellaneous bands, whether they were the target protein was judged according to the prestained protein marker. Two channels were used for the exposure, one for the marker (which can show the shape of the PVDF membrane and the marker's location) and one for the target band(if the antibody is effective or the expression of the target protein is high, after incubation with specific antibody, only one clear target band can be seen in black). We try to splice the cut PVDF membranes as the whole gels' shape before visualizing. Pictures were then exported to TIFF format for results display , and the alignment and annotation were completed on Adobe Illustrator. Red boxes are marked for the bands presented in the manuscript.

Figure 3a

Repeat 1

FOXO1
(70-80kDA)

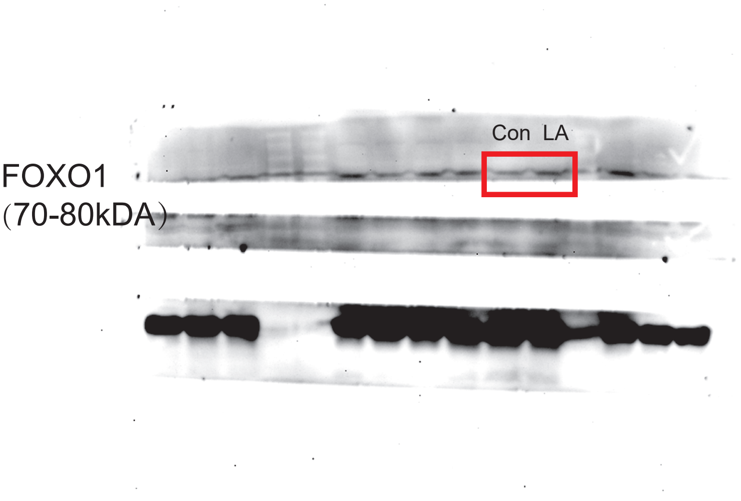

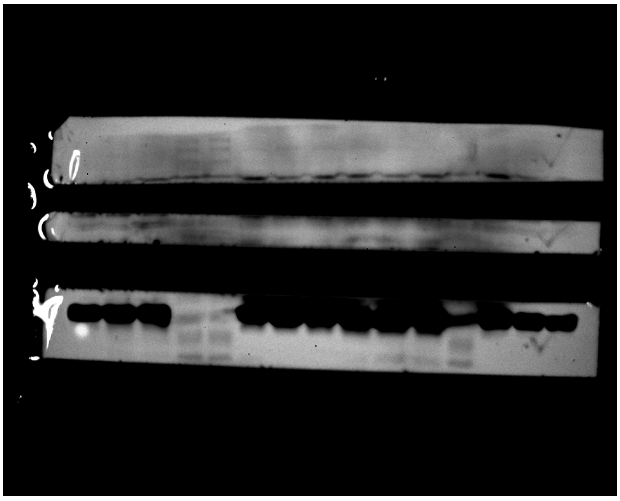

GAPDH
(36kDa)

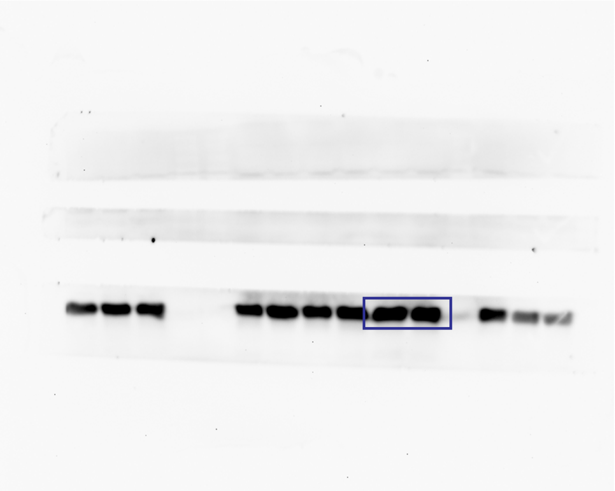

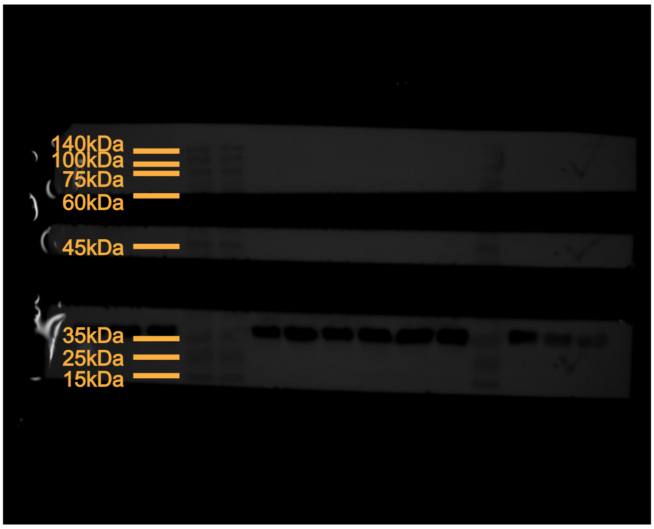

Repeat 2

FOXO1
(70-80kDA)
GAPDH
(36kDa)

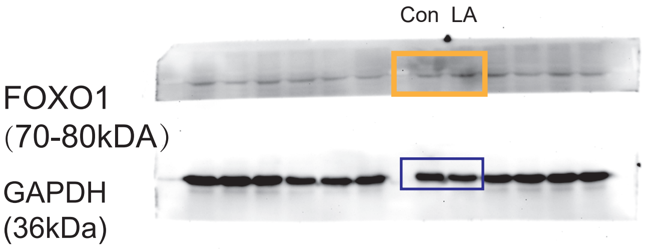

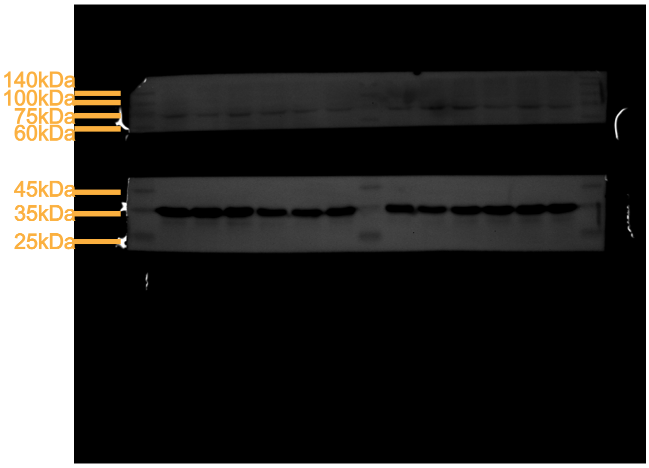

Repeat 3

FOXO1
(70-80kDA)

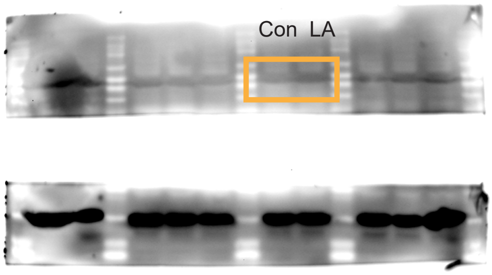

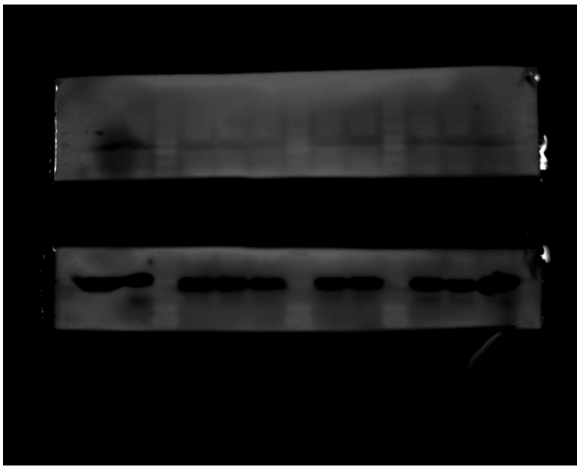

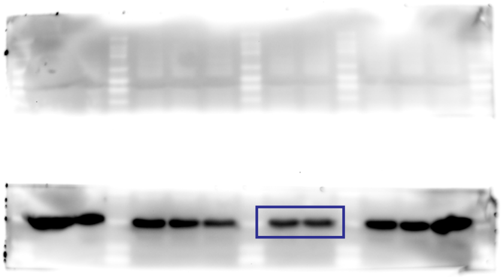

GAPDH
(36kDa)

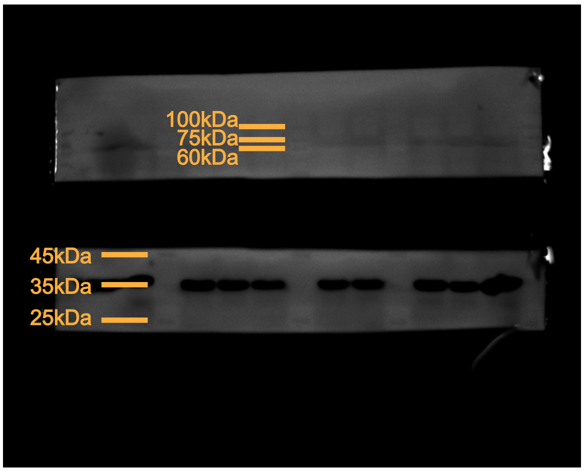

Fig 3f

repeat 1

FOXO1
(70-80kDA)

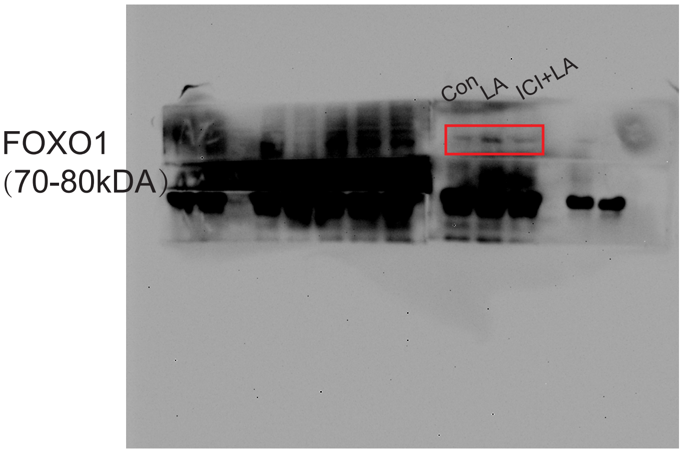

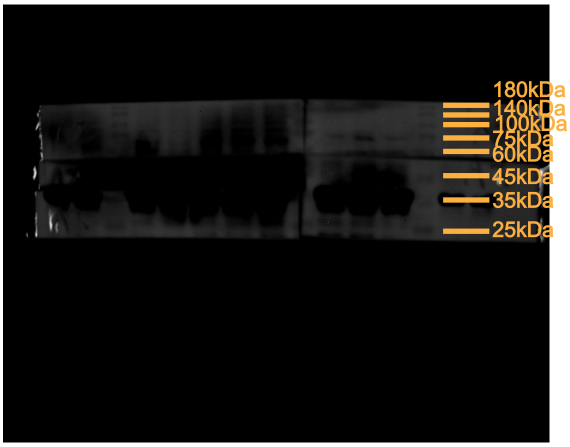

GAPDH
(36kDa)

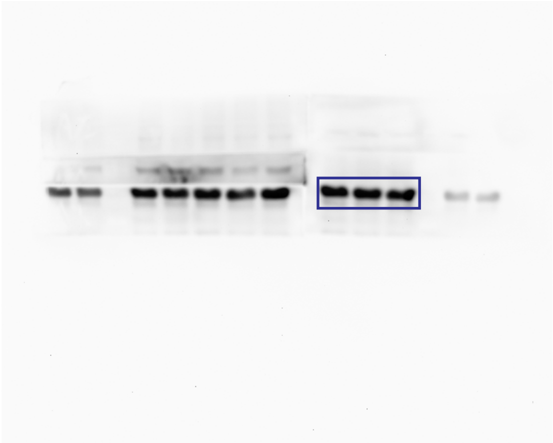

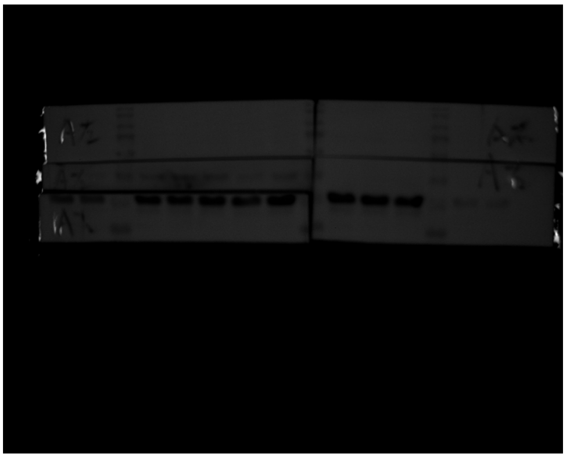

repeat 2

FOXO1
(70-80kDA)

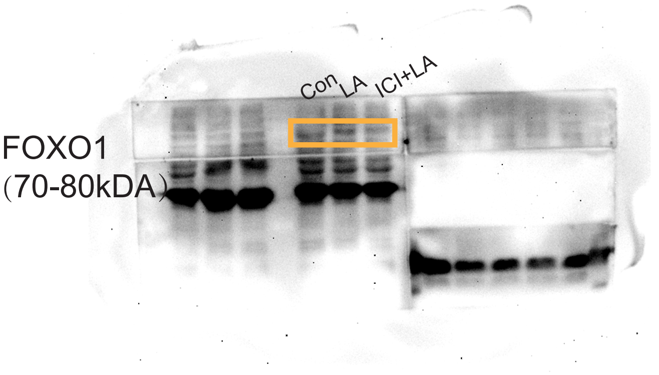

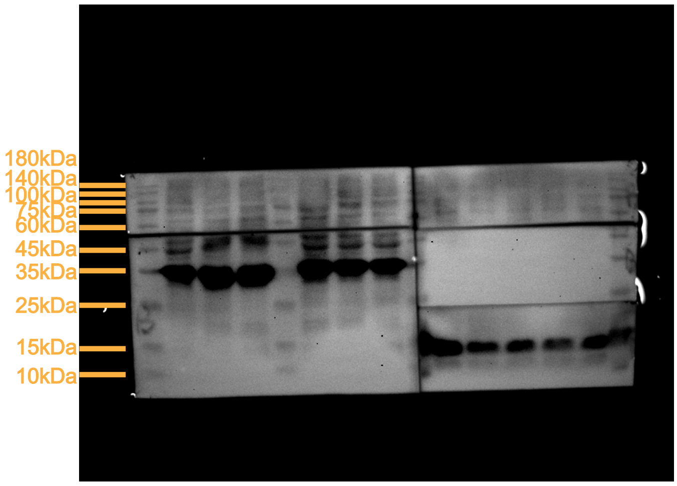

GAPDH
(36kDa)

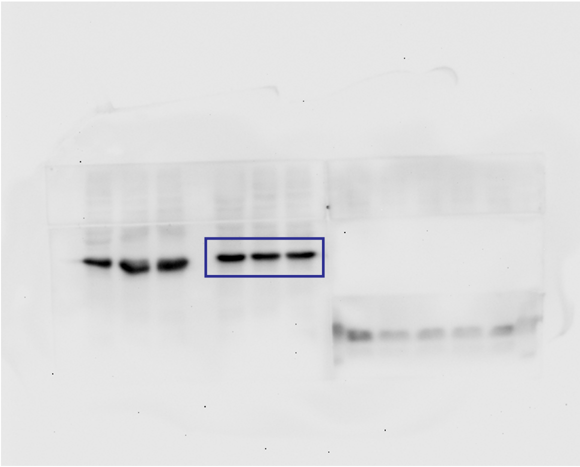

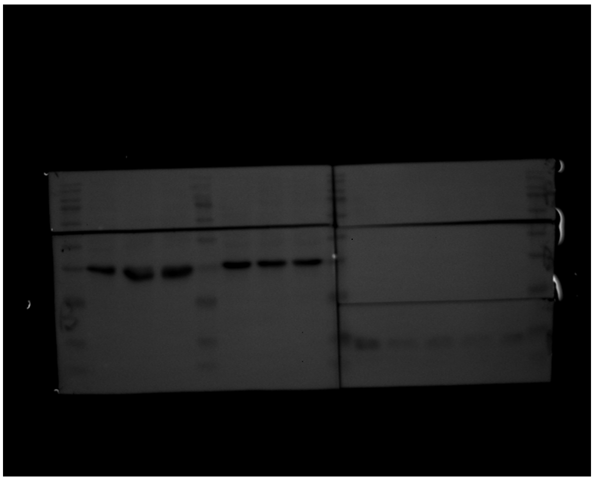

repeat 3

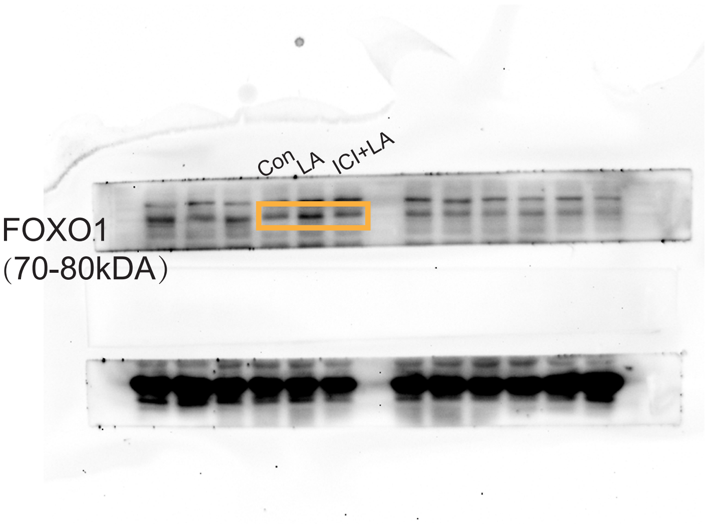

FOXO1
(70-80kDA)

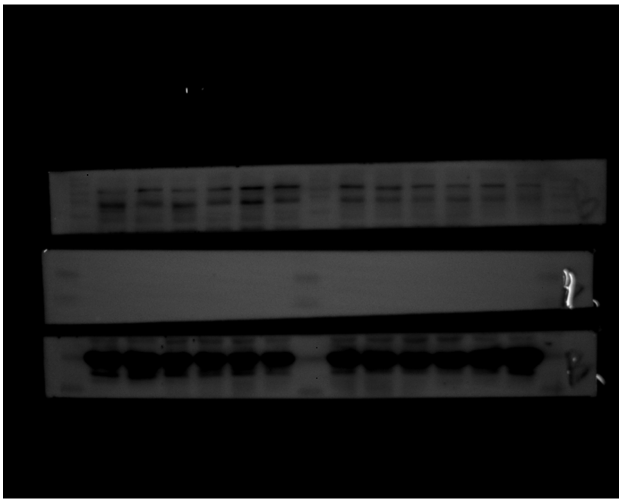

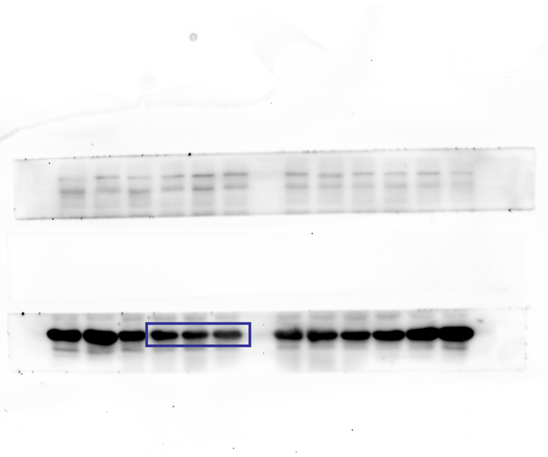

GAPDH
(36kDa)

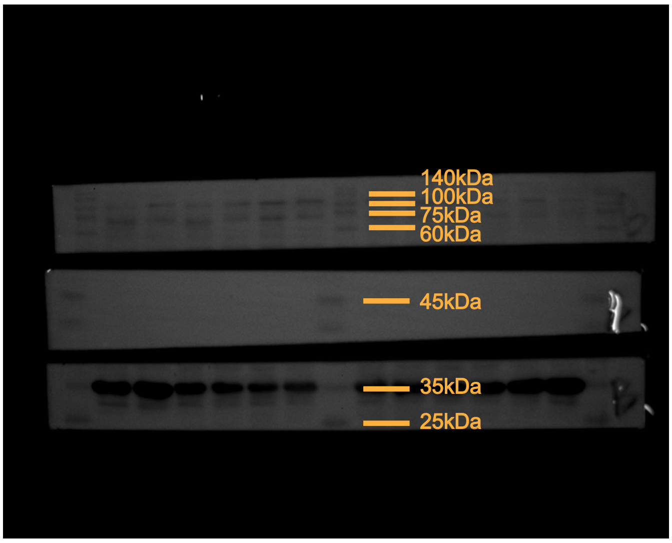

Fig 5f

P65 repeat 1

P65 (65KDa)

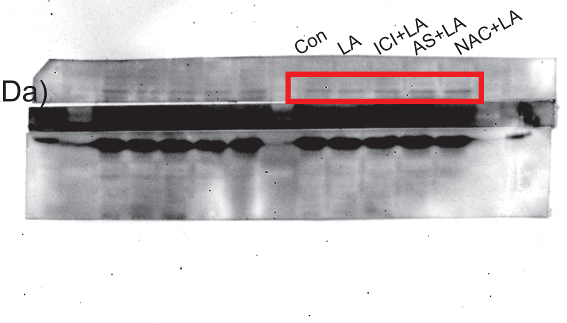

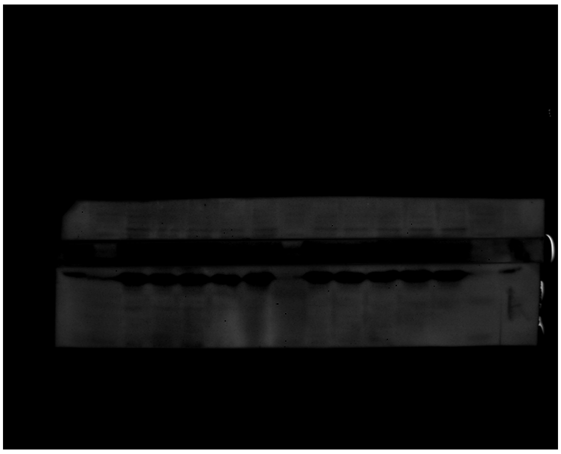

GAPDH (36kDa)

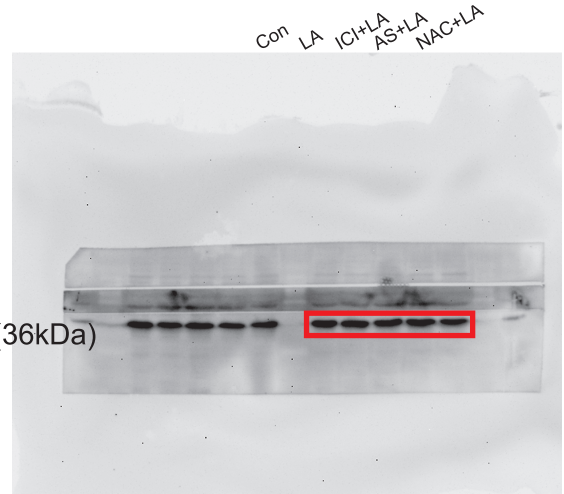

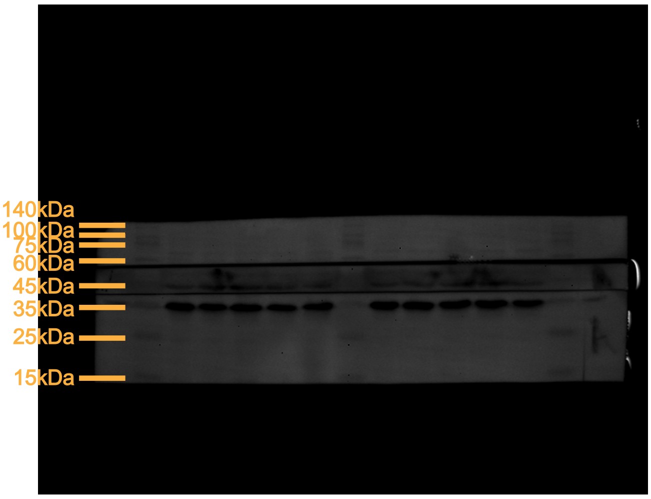

P65 repeat 2

P65 (65KDa)

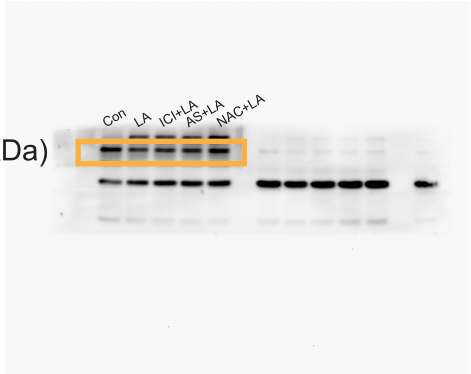

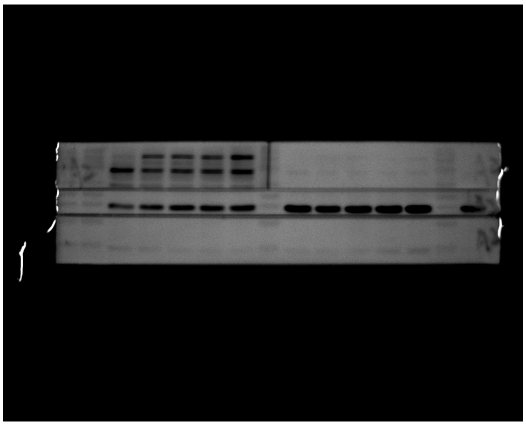

GAPDH (36kDa)

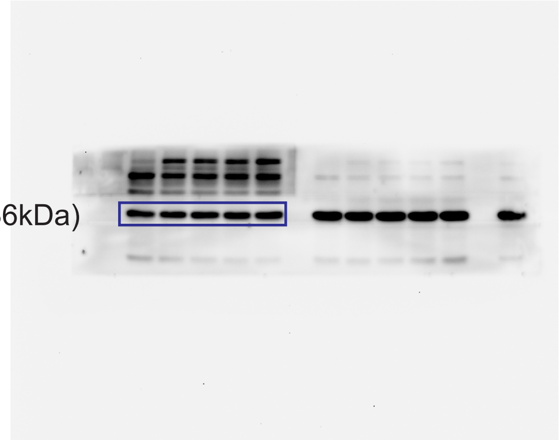

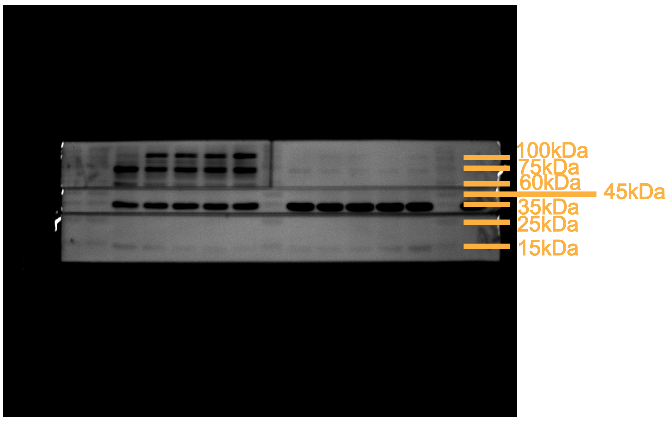

P65 repeat 3

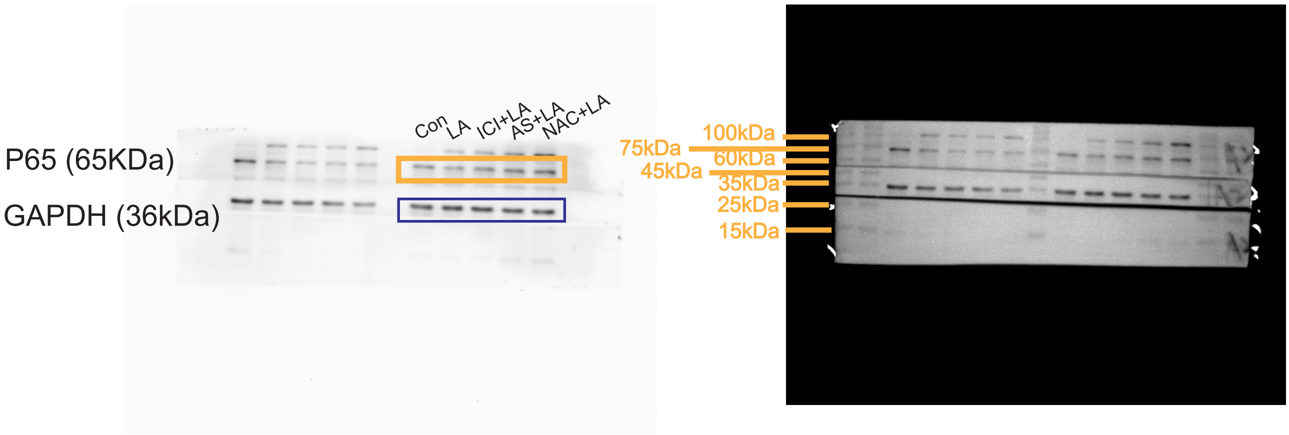

p-P65 repeat 1

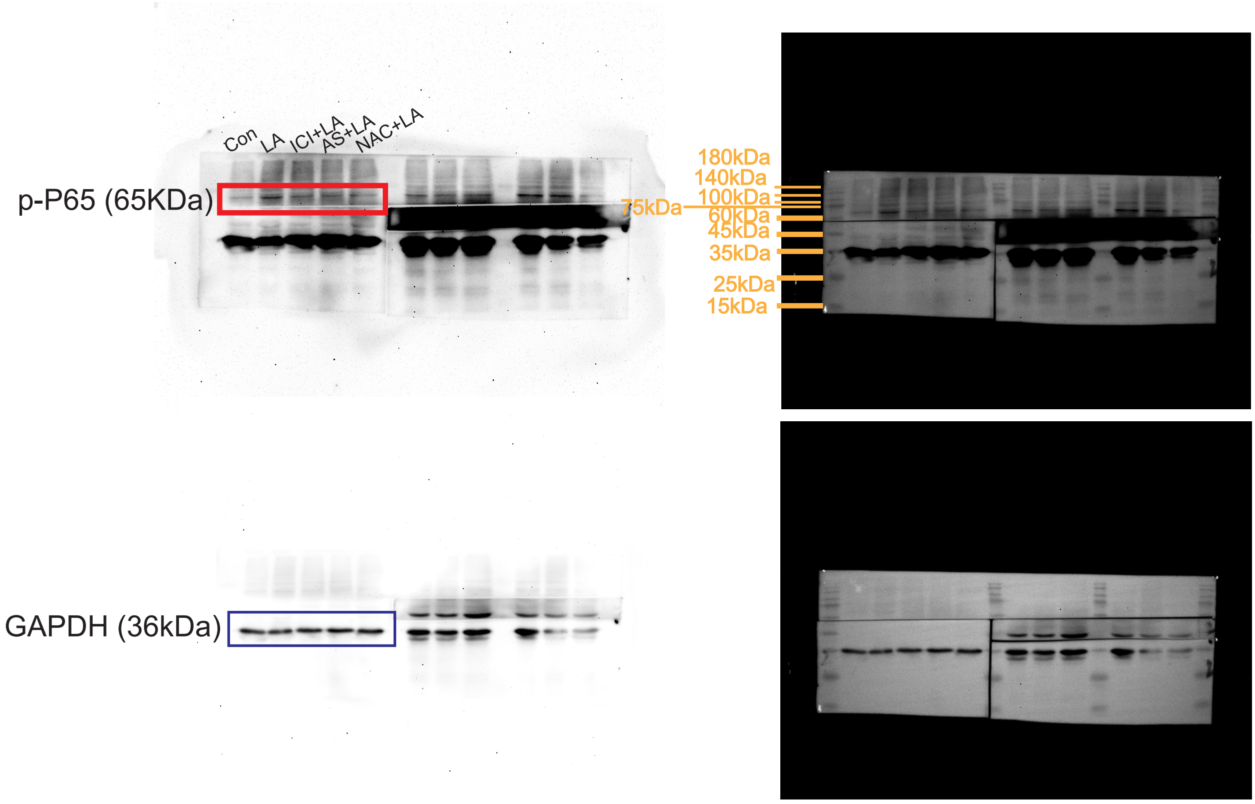

p-P65 repeat 2

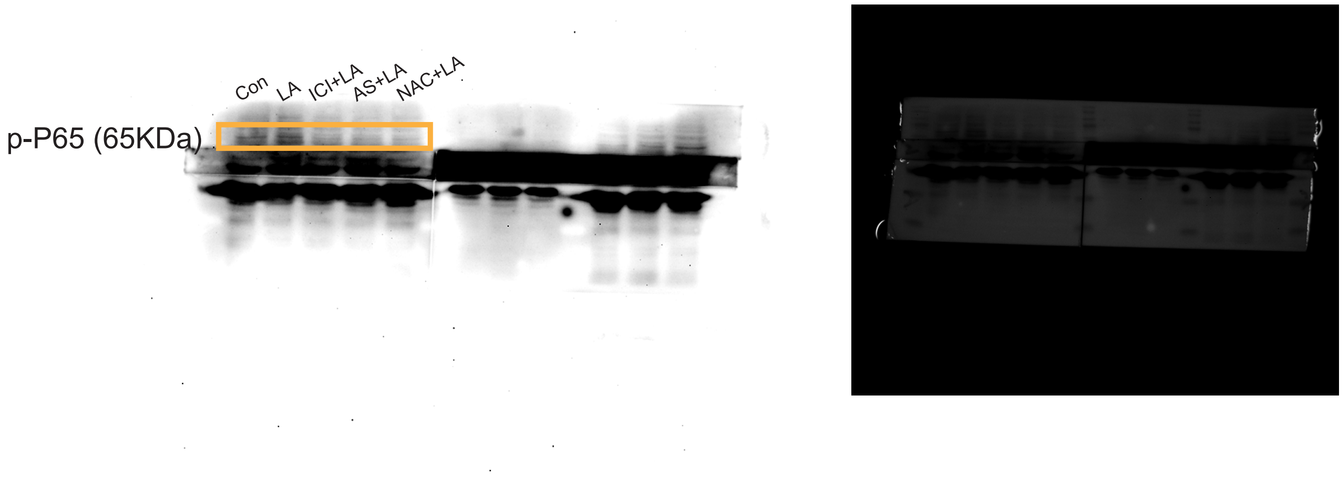

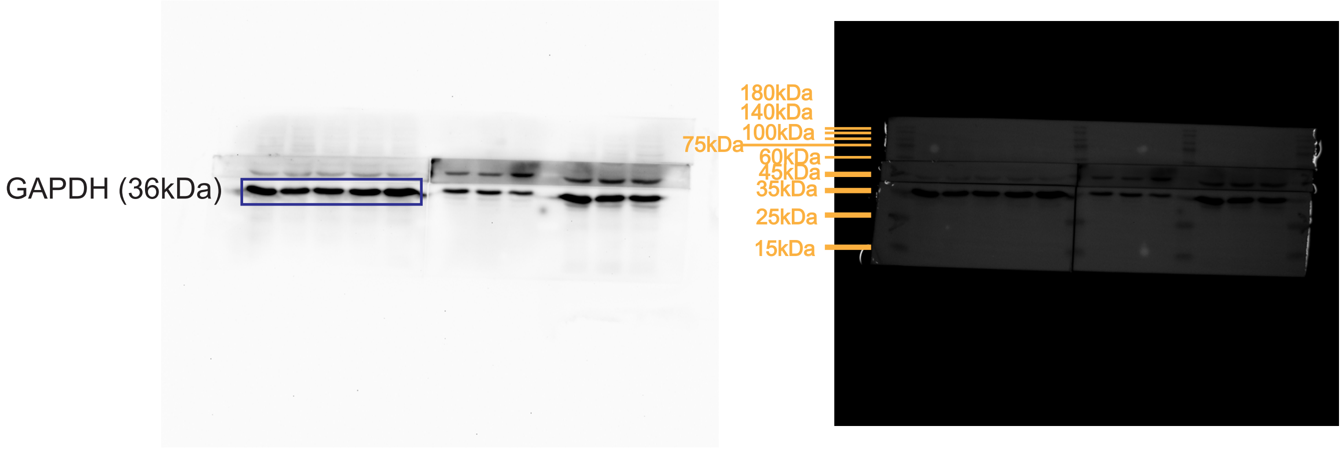

p-P65 repeat 3

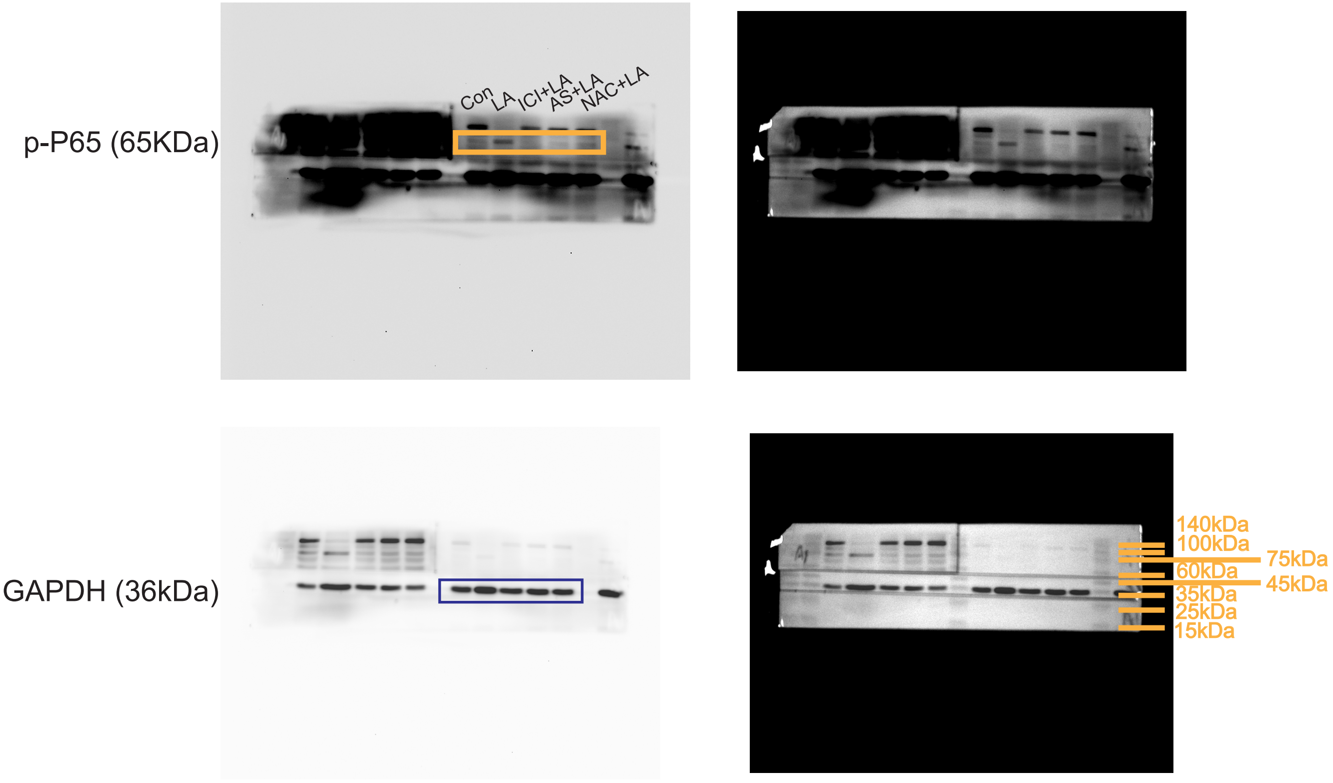

IκBα repeat 1

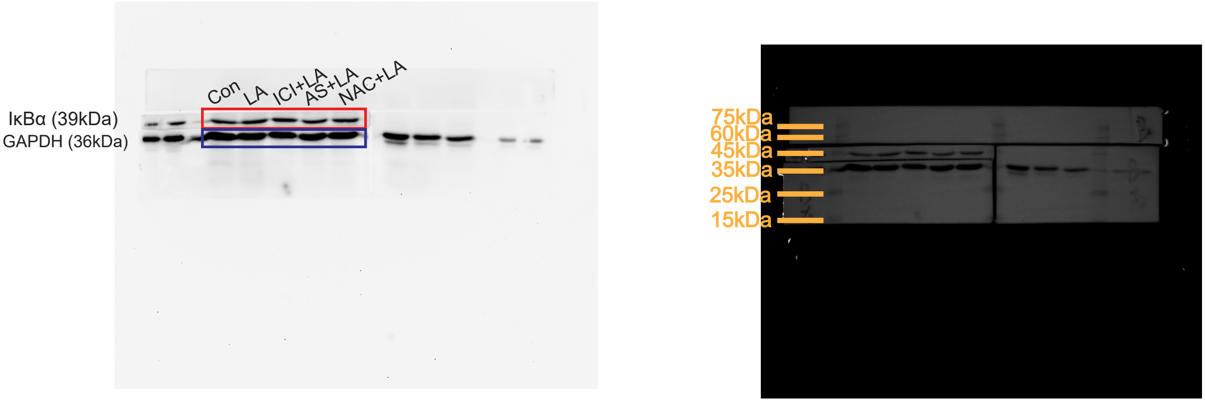

IκBα repeat 2

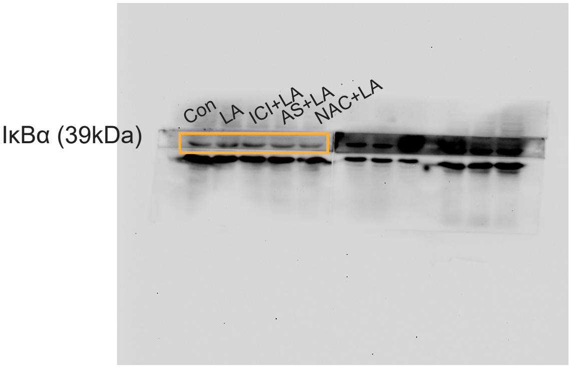

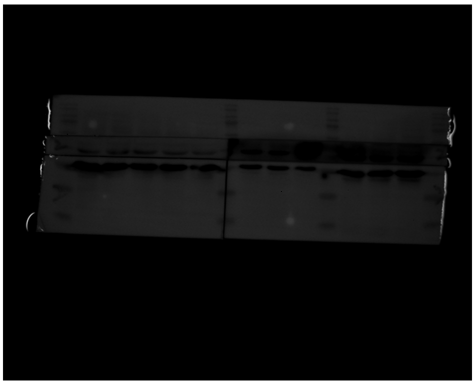

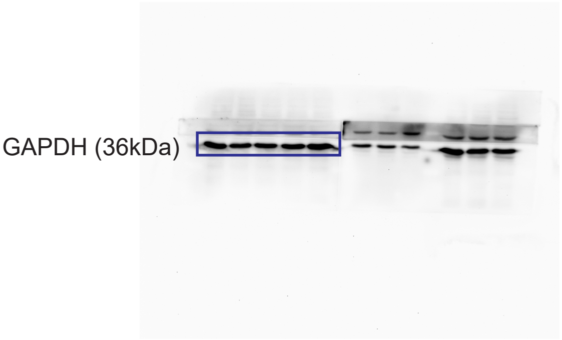

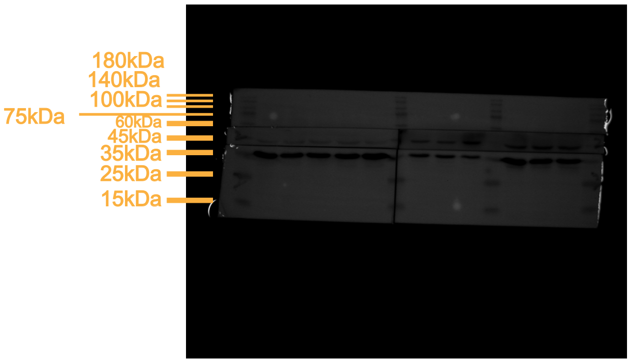

IκBα repeat 3

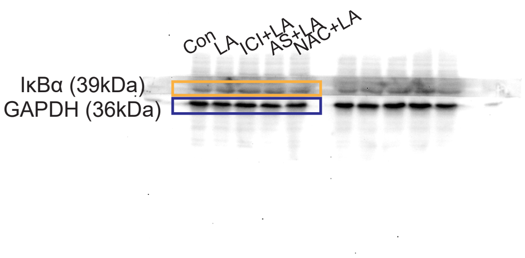

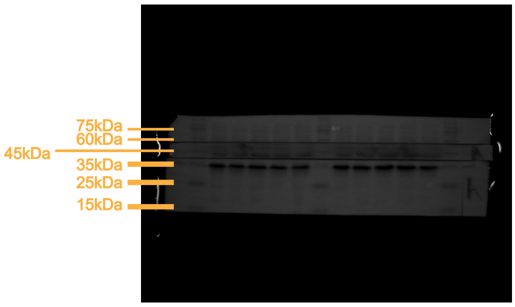

p-IκBα repeat 1

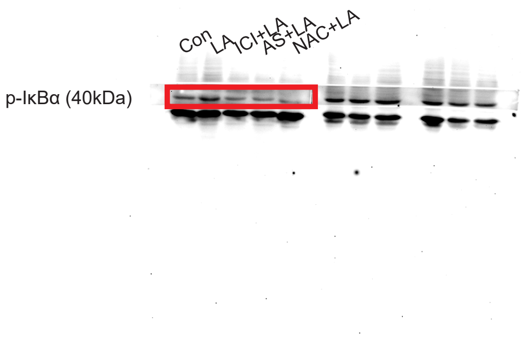

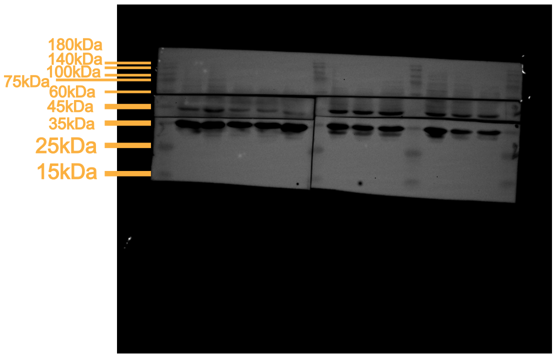

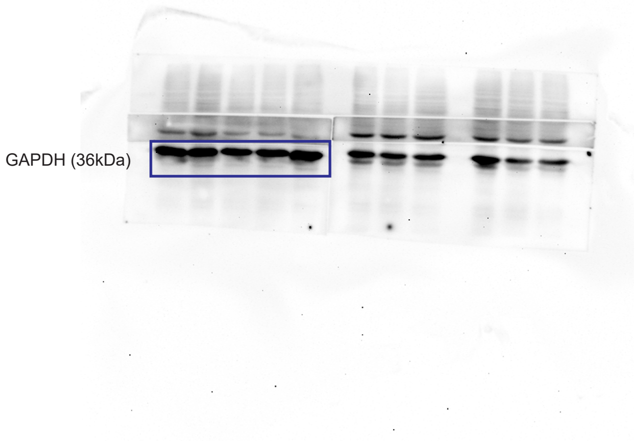

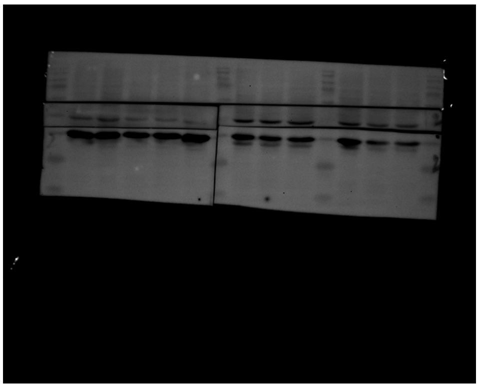

p-IκBα repeat 2

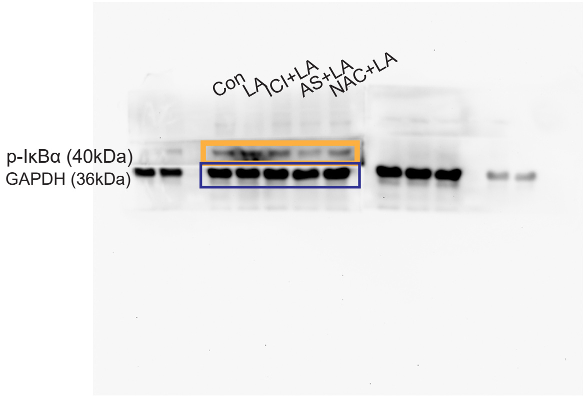

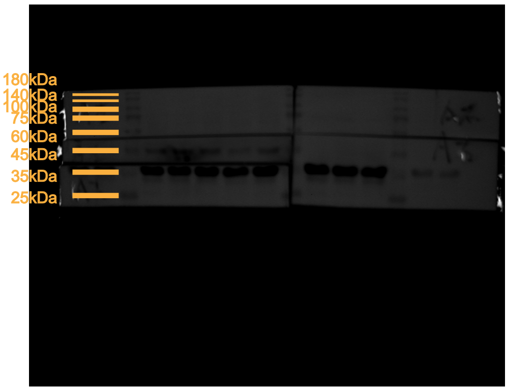

p-IκBα repeat 3

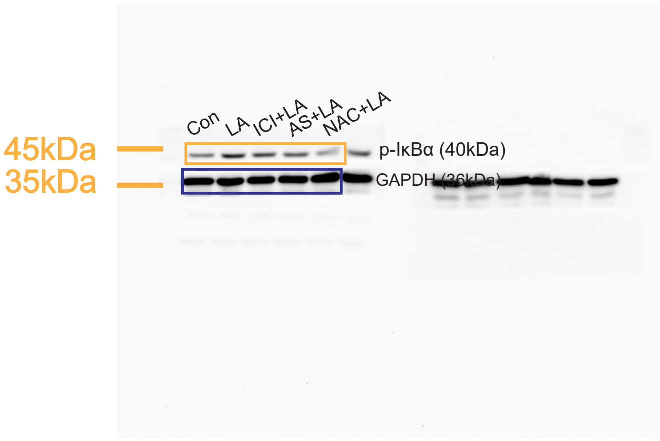

This repetition was due to the fact that we had just started learning how to conduct WB experiments and didn’t not understand the requirements for storing photos, so there is only one channel for photos.
